# Fractures of the capitellum humeri and their associated injuries

**DOI:** 10.1007/s11678-018-0441-9

**Published:** 2018-02-05

**Authors:** Valentin Rausch, Matthias Königshausen, Thomas A. Schildhauer, Jan Gessmann, Dominik Seybold

**Affiliations:** Department of General and Trauma Surgery, BG University Hospital Bergmannsheil, Bürkle-de-la-Camp-Platz 1, 44789 Bochum, Germany

**Keywords:** Humeral fracture, Humerus, Elbow joint, Classification, Radius fractures, Humerusfraktur, Humerus, Ellbogengelenk, Klassifikation, Radiusfrakturen

## Abstract

**Objective:**

Fractures of the capitellum are rare injuries but are often more complex and of a greater extent than assumed from conventional radiographs. Classification is usually based on their extension in relation to the trochlea the trochlea and on the number of fragments. Information on associated injuries is limited and only reported in small case series. The aim of this retrospective study was to report on our experience with capitellar fractures and their associated injuries.

**Methods:**

We retrospectively reviewed all patients treated for fractures of the capitellum humeri at our institute between 2005 and 2017. Fractures were classified according to the Bryan–Morrey and the Dubberley classification and analyzed for their associated injuries depending on the fracture type using the chi-squared test.

**Results:**

A total of 27 capitellar fractures were treated at our institute between 2005 and 2017. The median age of the patients was 57 years (range, 4–78) and they were all treated operatively. Associated injuries of the elbow were found in 12 cases (ten radial head fractures, two elbow dislocations, two tears of the radial collateral ligament). The injury mechanism was known for 26 patients (four fell on their outstretched arm, 19 suffered a direct blow to their elbow, two had a traffic accident).

**Conclusion:**

The incidence of radial head fractures is high in patients with capitellar fractures. Patients suffering a fracture of the capitellum humeri should be thoroughly examined for such associated injuries since a missed diagnosis can lead to poor outcomes.

## Zusammenfassung

**Ziel der Arbeit:**

Frakturen des Capitulum humeri sind seltene Verletzungen, die häufig eine komplexere Frakturmorphologie haben und weitreichender sind als nach konventionellen Röntgenaufnahmen zu vermuten. Die verschiedenen Klassifikationen dieser Fraktur basieren auf der Ausdehnung der Fraktur in Relation zur Trochlea humeri und der Anzahl der Fragmente. Daten über Begleitverletzungen dieser Fraktur sind in der Literatur nur begrenzt vorhanden und beschränken sich meist auf kleine Fallserien. In der vorliegenden Untersuchung berichten die Autoren über ihre Erfahrung in der Behandlung von Capitulumfrakturen und deren Begleitverletzungen.

**Methoden:**

Für diese Untersuchung wurden alle Patienten retrospektiv erfasst, die sich zwischen 2005 und 2017 mit einer Capitulumfraktur in der Klinik der Autoren in Behandlung befanden. Die Frakturen wurden erneut nach der Bryan-Morrey und der Dubberley-Klassifikation eingeteilt. Vorhandene Begleitverletzungen wurden einbezogen. Mithilfe des Chi-Quadrat-Tests wurden Begleitverletzungen mit dem Frakturtyp korreliert.

**Ergebnis:**

Insgesamt wurden 27 Frakturen des Capitulum humeri zwischen 2005 und 2017 mit einem Median von 57 Jahren (zwischen 4 und 78 Jahren) in der Klinik der Autoren behandelt. Bei allen Patienten erfolgte eine operative Versorgung. Begleitverletzungen wurden in 12 Fällen gefunden (10 Radiuskopffrakturen, 2 Ellenbogenluxationen, 2 Rupturen des radialen Kollateralbands). In 26 Fällen war der Unfallmechanismus erinnerlich (4 Stürze auf den ausgestreckten Arm, 19 direkte Anpralltraumata, 2 im Rahmen eines Verkehrsunfalls).

**Schlussfolgerung:**

Die Inzidenz begleitender Frakturen des Radiuskopfes ist im Rahmen von Capitulumfrakturen hoch. Bei der Diagnose einer Capitulumfraktur sollte daher sorgfältig nach möglichen Begleitverletzungen gesucht werden, da übersehene Frakturen zu einem ungünstigeren Ergebnis führen können.

## Introduction

Fractures of the capitellum humeri are rare injuries accounting for only 1% of all fractures and around 6% of fractures close to the elbow [15]. Hahn first described a fracture of the capitellum in 1853 [10]. Since then, several classifications have been developed for these fractures. The classifications most commonly used for capitellum fractures are the descriptive Bryan and Morrey classification (modified by McKee et al.) and the Dubberley classification [8, 13–15]. Another classification was proposed by Ring et al., generally focusing on coronal shear fractures of the distal humerus [19].

**Fig. 1:**
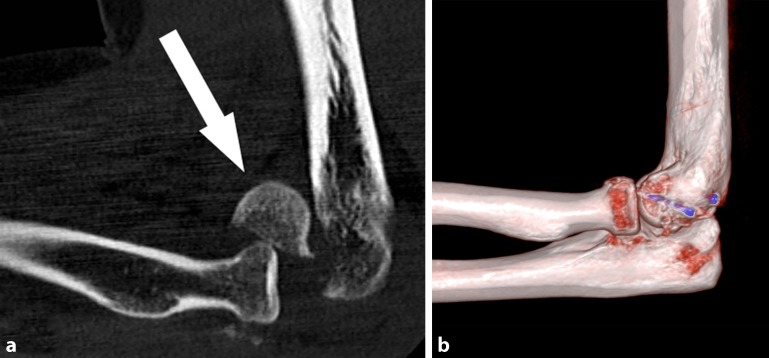
**a** Sagittal computed tomography reconstruction of the injured elbow. Shear fracture of the capitellum (type I) with associated anterior radial head injury (*arrow*). **b** After osteosynthesis with two headless screws (in posterior–anterior direction)

**Fig. 2:**
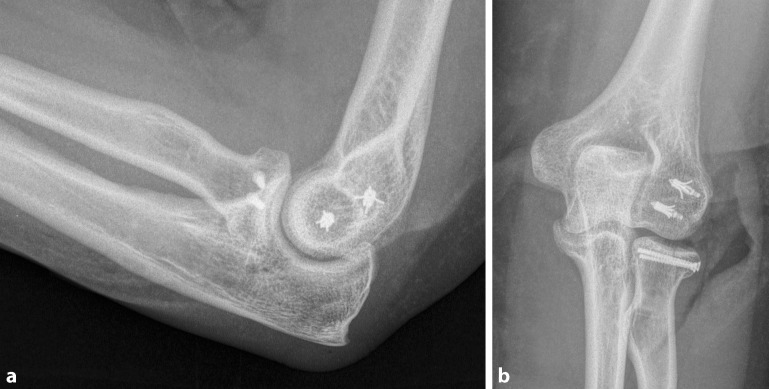
**a** Lateral and** b** anterior–posterior radiographs of the injured elbow. Capitellar fracture (type III) with associated Mason-II fracture of the radial head and tear of the lateral collateral ligament after operative treatment. The capitellar fracture was treated with bioabsorbable pins, torn ligaments were refixated using Mitek anchors, and the radial head fracture was osteosynthesized with two small-fragment screws

Today, capitellum fractures are usually treated by osteosynthesis. Single-fragment uncomminuted fractures (type I and IV according to the modified Reagan–Morrey classification) can be successfully treated using headless screws (Fig. 1), while fixation of smaller fragments can be achieved with bioabsorbable pins (type II; Fig. 2; [11, 12, 21, 22, 26]). Comminuted fractures (type III) are stabilized by applying an additional buttress plating to the fracture site [19].

Capitellum fractures are often more complex than expected upon analyzing conventional radiographs [19]. Computed tomography (CT) is therefore regularly recommended in these cases so as to diagnose the extent of the fracture and to plan operative treatment.

Since capitellar fractures are rare, the data on associated injuries in fractures of the capitellum are limited to smaller studies providing clinical data of these fractures. The aim of this study was to investigate the mechanism of injury, the treatment, and the injuries associated with capitellar fractures.

## Patients and methods

Patients with a partial intra-articular distal humerus fracture treated at our institute between 2005 and 2017 were identified by medical chart review. Available imaging studies, including conventional radiographs as well as CT scans, were reviewed. Patients with fractures of the capitellum were included in the study. Fractures involving the epicondyle or low-plane fractures of the distal humerus were excluded. Fractures were then selected and classified using the archived radiographs according to the modified Bryan–Morrey and the Dubberley classifications. All capitellar fractures treated at our institute with available imaging were included in this study comprising 27 patients.

Conventional radiographs, CT scans, and surgical reports were reviewed for associated lesions of the elbow joint. All patients had preoperative conventional radiographs of the elbow joint in anterior–posterior and lateral projection. For 21 patients, a preoperative CT scan was available, and for one patient an additional preoperative magnetic resonance imaging study could be reviewed. Ligamentous injuries were diagnosed intraoperatively. The frequencies of associated lesions and their distribution according to the fracture type were tested with the chi-squared test (95% confidence interval [CI]) using GraphPad Prism version 6.0c for Mac (GraphPad Software, La Jolla CA, USA).

## Results

We included 16 female patients and 11 male patients with a median age of 57 years (range, 4–78). Patients reported on the mechanism of their injury in 96% of cases (all but one case). Four patients fell on their outstretched arm, 19 patients suffered a direct blow to their elbow during a fall, and in two cases the injury was associated with a traffic accident on a bicycle (Table 1).

**Table 1:** Mechanism of injury

	Female		Male	
	*n*	Age, years (range)	*n*	Age, years (range)
Fall on outstretched arm	2	56.0 (45–57)	2	24.5 (19–30)
Direct blow to the elbow	12	62.4 (43–78)	7	39.4 (4–69)
Traffic accident	2	43.0 (43)	1	13
N/A	0	N/A	1	76
Total	16	59.1 (43–78)	11	37.6 (4–76)

*N/A* data not available

**Fig. 3:**
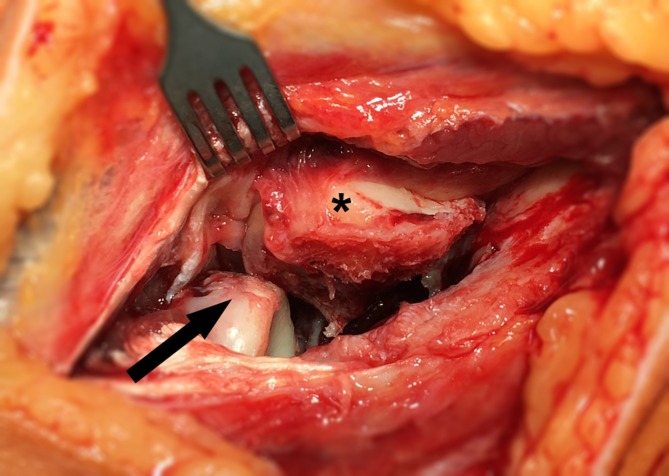
Shear fracture of the capitellum (type I) with associated anterior radial head injury (same case as in Fig. 1). The *arrow* indicates the injury on the anterior radial head, the *asterisk* indicates the shear fragment of the capitellum

Of the 27 fractures included in our study, 33% could be classified as a type I fracture according to the modified Bryan–Morrey classification (Table 2). The incidence of associated elbow injuries in capitellar fractures was 44% (12 cases; Table 3). Ten of the patients included in our study (37%) had an associated radial head fracture (Fig. 3). In two cases, a terrible triad injury (radial head fracture, posterior dislocation of the elbow joint, and coronoid fracture) was observed. A ligamentous injury to the radial collateral ligament was observed in two cases. The chi-squared test, used to analyze the distribution of associated lesions according to the fracture type (McKee or Dubberley), revealed no significant differences between capitellar fractures of a specific type.

**Table 2:** Clinical data and classification

	Female		Male		Total	%
	*n*	Age, years (range)	*n*	Age, years (range)		
Bryan–Morrey						
I	7	59.4 (43–78)	2	16.5 (4–29)	9	33
II	1	43	1	30	2	7
III	1	74	1	13	2	7
IV	7	59.1 (43–68)	7	48.3 (19–76)	14	52
Total	16	59.1 (43–78)	11	37.6 (4–76)	27	100
Dubberley						
1	9	59.2 (43–78)	9	31.1 (4–69)	18	67
2	5	59.1 (43–67)	2	67.0 (58–76)	7	26
3	0	–	0	–	0	N/A
N/A	2	55.5 (43–68)	–	–	2	7
Total	16	59.1 (43–78)	11	37.6 (4–76)	27	100

*N/A* data not available

**Table 3:** Associated injuries and classification

	Radial head or neck injury		Terrible triad		Ligamentous injury		No associated injury		Total
	*n*	%	*n*	%	*n*	%	*n*	%	*n*
McKee									
I	2	22	1	11	1	11	6	67	9
II	2	100	0	0	0	0	0	0	2
III	1	50	0	N/A	0	N/A	1	50	2
IV	5	36	1	7	1	7	8	57	14
Total	10	37	2	7	2	7	15	56	27
Dubberley									
1	6	33	2	11	2	11	10	56	18
2	2	29	0	0	0	0	5	71	7
3	0	N/A	0	N/A	0	N/A	0	N/A	0
N/A	2	100	0	0	0	0	0	0	2
Total	10	37	2	7	2	7	15	56	27

*N/A* data not available

All patients were treated operatively: ten patients were treated with Herbert screws (five in anterior–posterior orientation, five in posterior–anterior orientation). In general, screw fixation was performed on 17 patients, while plate fixation or a combination of screws, plates, or suture anchors was necessary in ten patients. Bioabsorbable pins were used in two cases. In one patient, a comminuted fracture of the capitellum could not be reconstructed and was therefore excised. Preoperative radiographs or CT scans were available for all patients.

Of the fractures, 26 healed in a timely manner. However, in one case, a comminuted fracture (Bryan–Morrey type III) resulted in necrosis of the capitellum.

## Discussion

Associated injuries occur frequently in capitellar fractures. We found injuries to the radial head in more than 37% of all capitellar fractures. Few studies exist providing clinical data of capitellar fractures. Watts et al. presented the largest number of cases [27]. They found a total of 19 radial fractures as well as two complex soft-tissue injuries (one nerve injury of the ulnar nerve in a McKee type IV fracture, one open type I fracture) in 79 partial intra-articular fractures of the distal humerus [27]. However, they could not find a correlation between the type of fracture and an associated injury. Other studies analyzing data on capitellar fractures mostly describe outcomes after specific types of osteosynthesis, such as outcomes after treatment with double-threaded screws, headless screws, cannulated screws, or bioabsorbable pins. There are only a few studies available describing the outcomes of nonoperative treatment or analyzing treatment in adolescents. In these studies, associated radial head injuries were found in up to 66% of type II capitellar fractures or 50% of type IV capitellar fractures (both according to the modified Bryan–Morrey classification; [9, 21]). Ruchelsman et al. were the first to report on the association of associated injuries in capitellum fractures with regard to outcome and specific fracture types [21]. In their cohort of 16 patients, they found five patients with associated radial head fractures. When comparing these two groups, patients with a radial head fracture had a slightly smaller arc of motion and worse functional scores (American Shoulder and Elbow Surgeons’ Score [ASES]: 35 vs. 39 points, Mayo Elbow Performance Index [MEPI]: 87 vs. 94 points). However, none of the clinical parameters in this comparison could be considered as statistically significant. The overall missing evidence of an association between radial head fractures and a specific fracture type might be due to the small number of fractures analyzed in the respective studies. This is also confirmed by the results of Watts et al., where no such association could be found in their study including 79 distal intra-articular shear fractures of the humerus [27].

The severity of intra-articular distal humeral fractures was first appreciated by Ring et al. [19]. They could only appreciate the complexity of the fractures upon surgical exposure during operative treatment and therefore recommend operative treatment with implants buried under the articular surface to restore function [19]. Operative treatment of capitellar fractures has also been shown to confer favorable clinical outcomes compared with nonoperative treatment and is therefore generally recommended in most cases [2, 4, 8, 9, 11–14, 18, 21, 22, 24–26]. A high incidence of associated injuries, especially of the radial head, may be explained as follows: fractures of the capitellum humeri frequently occur during a direct blow to the elbow or a fall on the outstretched arm, identical to the mechanism leading to a radial head fracture [1, 3, 16, 20]. In this scenario, associated radial head fractures (or capitellar fractures) can be viewed as corresponding lesions on the opposite site of the joint, a phenomenon that has been termed “kissing lesion” on the elbow by Claessen et al. [6]. This term is also used for opposing osteochondral lesions in other regions such as the talotibial joint [23]. In the elbow joint, however, this term has also been used for lateral osteochondral lesions caused by repetitive trauma [7].

Generally, cartilage injuries of the capitellum are known to be frequently present in patients with radial head fractures. Such lesions can be missed in preoperative imaging and may only be evident intraoperatively [5, 17]. Interestingly, Nalbantoglu et al. showed that higher grades of cartilage injuries are created by lower-grade radial head fractures, since the intact radial head can cause greater damage to the capitellum [17]. Consequently, both radial head and capitellum fractures, which in most cases result from patients falling on the outstretched arm, are regularly associated with related injuries of the corresponding joint.

### Limitations

The present study has certain limitations. First, owing to the low incidence of capitellar fractures, significant injuries associated with certain fracture types could not be found. Also, we only present the retrospective data acquired from patients treated for fractures of the capitellum. Differences in clinical outcomes of capitellar fractures with associated injuries at the elbow joint may reveal specific treatment strategies for these patients and need to be addressed in future studies.

## Practical conclusion

- The incidence of radial head fractures is high in patients with fractures of the capitellum humeri.
- Missed lesions of the radial head in patients with capitellar fractures may lead to inferior outcomes in such cases.
- A thorough inspection of the surrounding structures on radiographic images or intraoperatively is imperative, and fractures should be treated accordingly.
